# Preconditioning with Rapamycin Improves Therapeutic Potential of Placenta-Derived Mesenchymal Stem Cells in Mouse Model of Hematopoietic Acute Radiation Syndrome

**DOI:** 10.3390/ijms26104804

**Published:** 2025-05-17

**Authors:** Vasilii Slautin, Vladislav Ivanov, Alexandr Bugakov, Anna Chernysheva, Ilya Gavrilov, Irina Maklakova, Vladimir Bazarnyi, Dmitry Grebnev, Olga Kovtun

**Affiliations:** 1 Laboratory of Enteric Viral Infections, Federal Scientific Research Institute of Viral Infections «Virome», Federal Service for Surveillance on Consumer Rights Protection and Human Wellbeing, Yekaterinburg 620030, Russia; 2 Department of Pathophysiology, Ural State Medical University, Yekaterinburg 620014, Russia; vlanov123@gmail.com (V.I.); bugakov1999@mail.ru (A.B.); dr-grebnev77@mail.ru (D.G.); kovtun@usma.ru (O.K.); 3 Laboratory of Respiratory Viral Infections, Federal Scientific Research Institute of Viral Infections «Virome», Federal Service for Surveillance on Consumer Rights Protection and Human Wellbeing, Yekaterinburg 620030, Russia; chernysheva_ae@niivirom.ru; 4 Laboratory of Anti-Aging Technologies, Institute of Medical Cell Technologies, Yekaterinburg 620026, Russia; makliu@mail.ru; 5 Department of Physiology, Ural State Medical University, Yekaterinburg 620014, Russia; 6 Department of Medical Microbiology and Clinical Laboratory Diagnostics, Ural State Medical University, Yekaterinburg 620014, Russia; vlad-bazarny@yandex.ru

**Keywords:** mesenchymal stem cells, hematopoietic cytokines, preconditioning, acute radiation syndrome, rapamycin, autophagy

## Abstract

Acute radiation syndrome (ARS) results from high-dose ionizing radiation (IR) exposure, with bone marrow (BM) being highly susceptible due to its proliferative activity. BM injury causes pancytopenia, leading to infections, anemia, and bleeding. Mesenchymal stem cells (MSCs) hold promise for ARS treatment because of their immunomodulatory, anti-inflammatory, and regenerative properties. However, challenges such as replicative senescence, poor survival, and engraftment in irradiated microenvironments limit their efficacy. This study evaluated rapamycin-preconditioned placenta-derived MSCs (rPD-MSCs) in a mouse ARS model. Rapamycin was selected for preconditioning due to its ability to induce autophagy and modulate cytokine secretion. We assessed rapamycin-dependent modulation of autophagy-related genes and proteins, as well as hematopoietic cytokines secretion in PD-MSCs, and evaluated morphological changes in blood and BM at 7 and 21 days post-irradiation in ICR/CD1 mice. Preconditioning with rapamycin alters the secretion of granulocyte colony-stimulating factor (G-CSF), stem cell factor (SCF), and Fms-related tyrosine kinase 3 ligand (Flt3LG) in PD-MSCs without affecting cell viability. rPD-MSCs better enhance hematopoietic recovery, restore bone marrow cellularity, and increase peripheral blood cell counts by elevating the secretion of hematopoietic cytokines compared to non-preconditioned cells. These results highlight rapamycin preconditioning as a promising strategy to enhance MSCs therapeutic potential for ARS, supporting further preclinical and clinical exploration.

## 1. Introduction

Damage to healthy internal tissues or organs caused by ionizing radiation is a significant public health issue. This type of injury can occur in situations such as accidental nuclear or radiological emergencies, as well as during medical procedures like total body irradiation before bone marrow transplantation or the administration of medical radioisotopes for cancer therapy [1].

Acute radiation syndrome is a serious illness caused by exposure to high doses of IR, as defined by the US Center for Disease Control and Prevention (CDC). It happens when the body is exposed externally to a significant dose of radiation (typically ≥2 Gray (Gy)), involving penetrating forms of radiation such as high-energy X-rays, gamma rays, or neutrons, and when the entire body is affected [2,3]. ARS is characterized by four primary syndromes: hematopoietic (h-ARS), gastrointestinal (GI-ARS), cardiovascular (CV-ARS), and central nervous system acute radiation syndrome (CNS-ARS). h-ARS is defined by the loss of peripheral blood cells (pancytopenia) due to damage to hematopoietic stem cells in BM. This leads to the suppression of BM function and the development of secondary complications such as neutropenia and infections, anemia, and bleeding [4,5]. Given the severe and life-threatening nature of ARS, there is an urgent need to develop new and effective treatment methods to address this critical condition.

In this context, MSCs have emerged as a promising candidate because of their low immunogenicity, accessibility, ease of production, high proliferation rate, significant proliferative potential, and well-described immunomodulatory, anti-inflammatory, and regenerative properties [6,7,8,9]. Firstly described by A.J. Friedenstein in 1968 [10], MSCs are adult multipotent stem cells isolated in partially or fully differentiated prenatal, fetal, adult tissues, such as adipose tissue, bone marrow, placenta, umbilical cord, menstrual blood, dental pulp, and others [11]. According to the International Society for Cell and Gene Therapy (ISCT) criteria, MSCs should demonstrate the ability to differentiate into adipogenic, osteogenic, and chondrogenic lineages. Similar to MSCs isolated from human tissues, MSCs derived from mice are characterized by the expression of specific surface markers, including endoglin (CD105), integrin-β1 (CD29), hyaluronic acid receptor (CD44), and stem cell antigen-1 (Sca-1). Conversely, these cells lack the expression of markers such as pan-leukocyte antigen (CD45), platelet/endothelial cell adhesion molecule-1 (CD31), and lymphocytic antigen 76 (Ly76) [12,13].

Compared to other sources, MSCs from prenatal and fetal tissues demonstrate greater potential due to their enhanced proliferative capacity and activity, as well as therapeutic properties [14,15]. In this light, particularly regarding potential treatments for ARS, placenta-derived mesenchymal stem cells (PD-MSCs) represent a promising option because of their enhanced immunomodulatory properties, along with proliferative activity and potential comparable to other prenatal and fetal-derived sources [16].

Previous studies have shown that MSCs can support the growth of hematopoietic stem cells (HSCs) and their progeny by secreting various cytokines, including G-CSF, Flt3LG, and SCF [17]. Co-cultivation of MSCs with irradiated CD34+ cells promotes the growth of CD34+ cells, while their co-transplantation stimulates megakaryocytopoiesis in sublethally irradiated NOD/SCID mice [18,19]. During in vitro differentiation, MSCs adopted hematopoietic and endothelial characteristics, and their transplantation in lethally irradiated mice supported normal hematopoietic recovery, with donor cells transiently appearing in peripheral blood and lungs, while MSC-derived microvesicles provided similar protective effects as whole MSCs [20]. In the article by S. Shim et al., human umbilical cord blood-derived mesenchymal stem cells (hUCB-MSCs) were evaluated for their therapeutic potential in hematopoietic recovery following total body irradiation, demonstrating superior efficacy compared to G-CSF. hUCB-MSCs significantly enhanced leukocyte counts, modulated hematopoietic cytokines (Flt-3LG and TGF-β1), and promoted bone marrow regeneration [21]. K.X. Hu et al. investigated the effects and mechanisms of MSCs on hematopoietic reconstitution and bone marrow cell apoptosis in irradiated mice, demonstrating that MSCs infusion accelerated blood cell recovery, reduced apoptosis, improved cell cycle progression, and enhanced colony-forming unit activity, while also improving survival after 8 Gy total body irradiation, suggesting MSCs as a potential alternative or adjunct to hematopoietic stem cells transplantation for treating acute radiation syndrome [22]. It has been previously demonstrated that systemically administered MSCs counteract inflammatory responses, support detoxification and stress management post-irradiation exposure, and rescue endogenous hematopoiesis through the release of trophic factors and modulation of the HSC niche, highlighting their potential as a rapid and effective first-line treatment for radiation-induced hematopoietic failure [23].

Key challenges, including replicative senescence and poor survival in irradiated microenvironments, significantly reduce the therapeutic potential of MSCs for ARS treatment. Different methods, such as preconditioning cells prior to transplantation, emerge as a promising strategy to address these limitations.

As a result of LPS preconditioning, MSCs produced exosomes with elevated levels of cytokines and growth factors (IL-6, IL-10, IL-15, IDO, and FGF-2), which significantly improved survival, reduced clinical scores and weight loss, and enhanced hematopoietic recovery in mice when administered 4 h after lethal irradiation [24]. Transfection with CXCR4 increased the engraftment of transplanted human umbilical cord mesenchymal stem cells (HUMSCs) in radiation-injured lung tissues. CXCR4-overexpressing HUMSCs not only ameliorated histopathological damage but also reduced radiation-induced expression of SDF-1, TGF-β1, α-SMA, and collagen I, while preventing the radiation-induced downregulation of E-cadherin [25].

Given rapamycin’s promising ability to modulate autophagy through an mTOR-dependent mechanism, this study investigates the therapeutic potential of rapamycin-preconditioned placenta-derived mesenchymal stem cells (PD-MSCs) in a mouse model of acute radiation syndrome, focusing on their ability to enhance hematopoietic recovery and mitigate radiation-induced damage in BM.

## 2. Results

### 2.1. PD-MSCs Identification and Differentiation by ISCT Criteria

**Figure 1:**
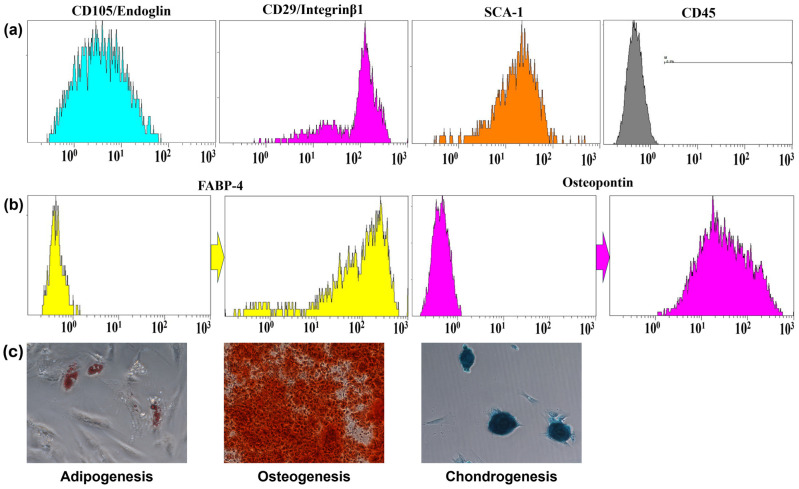
Identification of placenta-derived mesenchymal stem cells. (**a**) Analysis of CD105, CD29, CD45 and SCA-1 surface marker expression; (**b**) Results of PD-MSCs adipogenic and osteogenic differentiation evaluating by surface marker FABP-4 and Osteopontin expression using flow cytometry; (**c**) Adipogenic differentiation (Oil Red O staining); Osteogenic differentiation (Alizarin Red staining); Chondrogenic differentiation (Toluidine Blue staining). Magnification 200×.

The adhesive properties of PD-MSCs were confirmed during cultivation. Immunophenotypic analysis demonstrated the presence of characteristic surface markers (CD105, CD29, and SCA-1) and the lack of expression of the pan-leukocyte marker CD45 (Figure 1a). Adipogenic differentiation of PD-MSCs revealed a subset of cells containing small lipid droplets or not containing them, which was further validated by flow cytometry through the detection of fatty acid-binding protein 4 (FABP-4) expression. Furthermore, all cells within the culture underwent successful osteogenic differentiation, as evidenced by the expression of osteopontin (Figure 1b,c). In summary, the isolated cell culture exhibits characteristics consistent with MSCs and fulfills the International Society for Cellular Therapy (ISCT) criteria for MSCs.

### 2.2. Rapamycin-Dependent Modulation of Autophagy and Hematopoietic Cytokines Secretion in PD-MSCs

**Figure 2:**
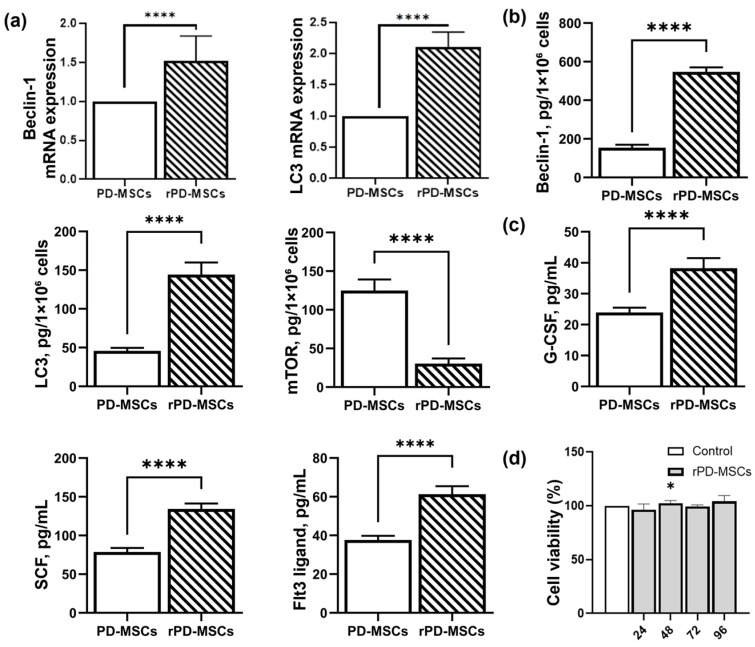
Rapamycin-dependent modulation of autophagy and hematopoietic cytokines secretion in PD-MSCs. (**a**) The level of BECLIN1 and LC3 genes expression; (**b**) The level of Beclin-1, LC3 and mTOR proteins in MSCs lysate; (**c**) The level of hematopoietic cytokines (G-CSF, SCF, Flt3LG) in the culture media; (**d**) Cell viability (%) by CCK-8 analysis; ****—*p* < 0.0001, *—*p* < 0.05.

Twenty-four hours after the addition of rapamycin, the expression of Beclin-1 and LC3 genes increased significantly compared to non-preconditioned cells (Figure 2b). Similarly, the levels of Beclin-1 and LC3 proteins in rPD-MSCs lysates were higher, while the level of mTOR protein was significantly reduced compared with PD-MSCs (Figure 2c). Rapamycin treatment also increased the secretion of hematopoietic cytokines (G-CSF, SCF, and Flt3L) in the culture media, as assessed by ELISA. No negative changes in cell viability were observed after 24, 48, 72, or 96 h of preconditioning with 3 µM of rapamycin, as measured using the cell counting kit-8 (CCK-8) assay.

### 2.3. Rapamycin-Mediated Regulation of Hematopoietic Cytokines (G-CSF, SCF, Flt3LG) Secretion in a Mouse Model of h-ARS

**Figure 3:**
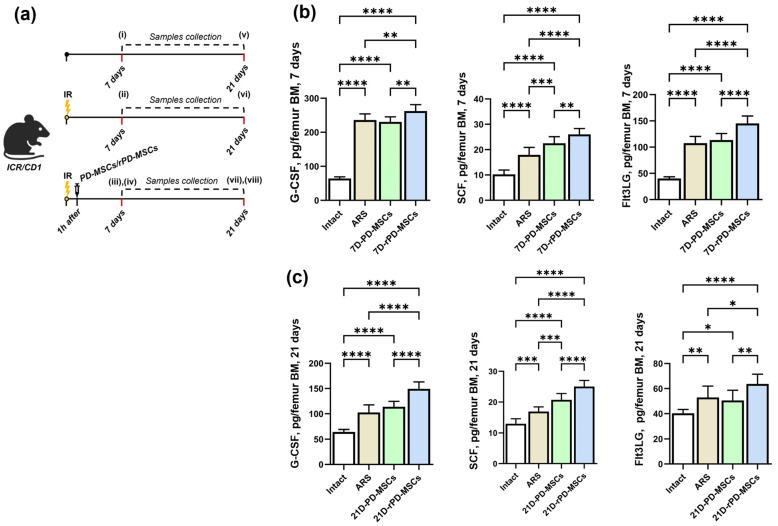
Levels of hematopoietic cytokines (G-CSF, SCF, Flt3 ligand) in mouse femur bone marrow at 7 and 21 days post-irradiation. (**a**) the distribution of animals by experimental groups and in vivo section of study design; (i–viii)–the number of experimental groups; (**b**) the levels of hematopoietic cytokines at 7 days post-irradiation; (**c**) the levels of hematopoietic cytokines at 21 days post-irradiation; ****—*p* < 0.0001, ***—*p* < 0.0005, **—*p* < 0.005, *—*p* < 0.05.

To confirm that preconditioning with rapamycin enhances hematopoietic cytokine secretion in PD-MSCs and that this effect persists after cell transplantation, levels of G-CSF, SCF, and Flt3L were evaluated in mouse femur bone marrow at 7 (Figure 3a) and 21 days post-irradiation (Figure 3b). Cytokine levels in the extracellular fraction of the BM were compared across eight groups. After irradiation, the levels of hematopoietic cytokines in the ARS group were significantly increased compared to the Intact group. No statistical differences were observed between the PD-MSC group and the ARS group in the levels of G-CSF and Flt3LG. However, the levels of G-CSF, SCF, and Flt3LG were significantly higher in the rPD-MSC group compared to the other groups at both 7 and 21 days post-irradiation. Notably, the concentrations of all cytokines in each group decreased by 21 days post-irradiation compared to levels at 7 days.

### 2.4. Rapamycin-Preconditioned PD-MSCs Enhance Hematopoietic Recovery, Restore Bone Marrow Cellularity in a Mouse Model of h-ARS

**Figure 4:**
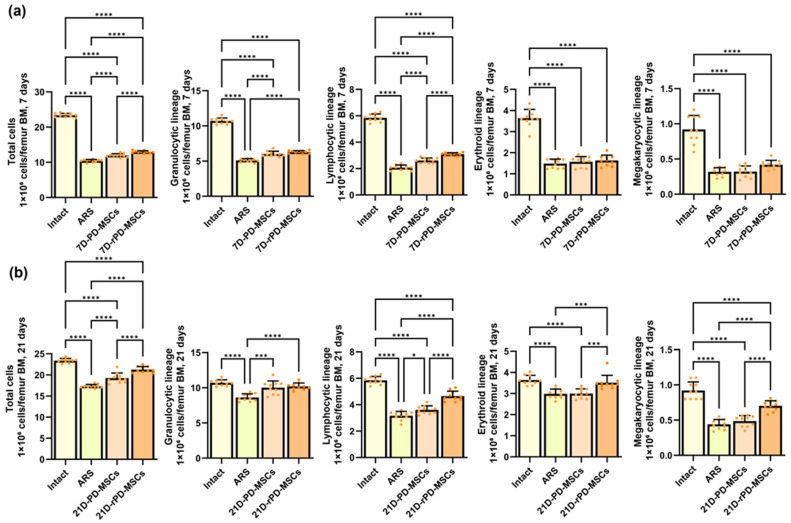
The bone marrow cellularity in mouse femur at 7 (**a**) and 21 (**b**) days post-irradiation. ****—*p* < 0.0001, ***—*p* < 0.0005, *—*p* < 0.05.

Bone marrow cellularity in mouse femurs was evaluated to assess the effects of PD-MSCs and rapamycin-preconditioned PD-MSCs at 7 and 21 days post-irradiation. At 7 days post-irradiation, all hematopoietic lineages were significantly depressed in the ARS group, consistent with the expected effects of radiation-induced bone marrow suppression (Figure 4a). By 21 days, minor increases in bone marrow cellularity were observed across all lineages, suggesting potential recovery processes (Figure 4b).

Transplantation of PD-MSCs resulted in increased cellularity in most hematopoietic lineages at both 7 and 21 days compared to the ARS group, with the exception of the erythroid lineage at 7 days and the megakaryocytic lineage at 21 days. Rapamycin preconditioning led to higher cellularity in all lineages at both time points compared to the ARS group, except for the erythroid lineage at 7 days. Furthermore, compared to the PD-MSC group, cellularity in the rPD-MSC group was higher in the total number of cells and the lymphocytic lineage at 7 days post-irradiation and in all lineages at 21 days post-irradiation, excluding the granulocytic lineage.

### 2.5. Rapamycin-Preconditioned PD-MSCs Significantly Increase Peripheral Blood Cell Counts Compared to Non-Preconditioned Cells in Irradiated Mice

Peripheral blood cell counts 7 days post-irradiation were characterized by pancytopenia, with the most significant loss observed in lymphocytes. By 21 days post-irradiation, positive changes in peripheral blood cell counts were observed, likely due to potential recovery processes. The number of monocytes in the 21D-ARS group showed no statistically significant difference compared to the Intact group.

PD-MSC transplantation leads to increased lymphocyte counts at 7 days post-irradiation and positive changes in leukocytes, lymphocytes, and granulocytes at 21 days post-irradiation. The level of monocytes in the PD-MSC group did not differ significantly from the Intact group.

**Figure 5:**
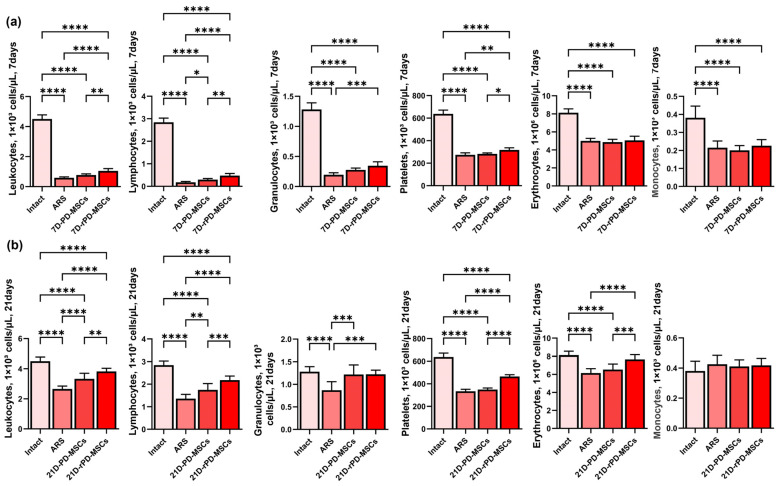
Analysis of peripheral blood cell count (leukocytes, lymphocytes, granulocytes, platelets, erythrocytes, and monocytes) at 7 (**a**) and 21 (**b**) days post-irradiation. ****—*p* < 0.0001, ***—*p* < 0.0005, **—*p* < 0.005, *—*p* < 0.05.

Rapamycin-preconditioned PD-MSCs highly increase the number of peripheral blood cells compared with non-preconditioned cells. At 7 days post-irradiation, the level of leukocytes, lymphocytes, and platelets were higher than in the ARS group and PD-MSC group, while granulocytes were higher only compared to the ARS group (Figure 5a). By 21 days post-irradiation, leukocytes, lymphocytes, platelets, erythrocytes, and granulocytes were increased in the rPD-MSC group compared to the ARS group. Furthermore, leukocyte, lymphocyte, platelet, and erythrocyte counts were higher in the rPD-MSC group compared to the PD-MSC group. Monocyte levels in the rPD-MSC group did not differ significantly from the Intact group (Figure 5b).

## 3. Discussion

High-dose IR exerts detrimental systemic effects on multiple organs and tissues, including the reproductive system, gastrointestinal tract, central nervous system, respiratory system, and cardiovascular system, among others [2,3]. The most life-threatening consequences arise from damage to the highly radiosensitive BM and hematopoietic system, which are particularly vulnerable to high-dose IR (typically ranging from 2 to 10 Gy) due to their high proliferative activity. A severe complication of IR exposure is h-ARS, characterized by the irreversible loss of the hematopoietic system’s regenerative capacity [4,5]. Nevertheless, survival outcomes following irradiation can be significantly enhanced through timely interventions, such as fluid transfusion, antimicrobial therapy, administration of molecularly cloned hematopoietic growth factors and cytokines, stem cell-based therapies, and other supportive treatments [26,27,28,29].

Previous studies have shown the efficacy of MSCs transplantation because of their ability to promote the growth of CD34+ cells, repair the bone marrow microenvironment, support hematopoiesis, and accelerate the recovery of peripheral blood cells [18,22,30,31]. Despite the high potential of MSCs in treating h-ARS, several challenges remain. For example, the harsh microenvironment in irradiated BM can reduce the regenerative ability of MSCs.

In this study, we evaluated the possibility of using PD-MSCs as a representative of MSCs from prenatal and fetal tissues, which demonstrate great advantages compared with MSCs from adult tissue, including better immunomodulatory properties [15,16]. During the adipogenic differentiation of PD-MSCs, we found a small population of cells containing small lipid droplets or lacking them, after successful immunophenotyping according to ISCT criteria. These cells were subsequently validated by flow cytometry through the detection of FABP-4 expression. It has been previously reported that MSCs derived from prenatal and fetal tissues exhibit low adipogenic differentiation potential but high chondrogenic and osteogenic potential, which is associated with the level of replicative senescence in cell culture [16]. Furthermore, to enhance the therapeutic potential of PD-MSCs for ARS treatment, we preconditioned them using rapamycin to increase secretion of hematopoietic cytokines by inducing mTOR-dependent autophagy.

Rapamycin modulates autophagy by inhibiting the mTOR/AKT signaling pathway through binding to the FK506-binding protein (FKBP12)-mTOR complex, thereby suppressing mTOR kinase activity. This initiates autophagy via Beclin-1 phosphorylation, promoting autophagosome maturation from the endoplasmic reticulum. Previous studies have shown that rapamycin does not affect MSC viability [32,33,34]. Furthermore, rapamycin preconditioning enhances MSC survival, improves MSC homing in damaged tissue, increases immunoregulatory properties, and inhibits proinflammatory cytokine secretion. These advantages allow the use of rapamycin-preconditioned MSCs for different pathological treatments. For instance, rapamycin-preconditioned MSCs were used for Cis-induced acute renal injury treatment [35], for the treatment of acute graft-versus-host disease [36], for recovery of infarcted myocardium [37], for multiple sclerosis treatment [38], and others. Following 24 h rapamycin preconditioning, we observed upregulated gene expression of Beclin-1 and LC3 without significant changes in cell viability. The level of these proteins in the MSC lysate has similar dynamics. The level of mTOR decreased.

We also evaluate the impact of rapamycin on hematopoietic cytokines (G-CSF, SCF, Flt3LG) secretion, because they play an important role in post-irradiation BM recovery. It has been previously shown that G-CSF, granulocyte-monocyte colony-stimulating factor (GMCSF), pegylated G-CSF (pegfilgrastim), interleukin-11, interleukin-3, and erythropoietin have regenerative effects on pancytopenia [31]. G-CSF, as a late-acting hematopoietic cytokine, has already been approved as an emergency treatment as an investigational new drug (IND) by the Centers for Disease Control and Prevention [28]. Additionally, Flt3LG, an early-acting cytokine, exhibits strong radioprotective effects against total-body irradiation in rabbits [39]. MSCs can also support the expansion of irradiated CD34+ cells in vitro when combined with SCF, Flt3LG, TPO, and IL-3, as shown by Mourcin et al. [18].

In this article, we evaluated the levels of Flt3LG, G-CSF, and SCF, due to their different roles in BM recovery. Our results demonstrate that preconditioning with rapamycin significantly increases the levels of these hematopoietic cytokines in culture media compared to non-preconditioned cells.

To determine whether the effects of in vitro rapamycin preconditioning on hematopoietic cytokine secretion persisted in vivo, we measured cytokine levels in the BM of mouse femurs at 7 and 21 days post-irradiation. We observed that, 7 days after irradiation and PD-MSC transplantation, there were no statistically significant differences in the levels of G-CSF and Flt3LG in ARS and PD-MSC groups. However, in the rPD-MSC group, the levels of all cytokines have significantly increased. Similar results were observed 3 weeks after irradiation and cell transplantation. It is important to highlight that cytokine levels were reduced to a similar extent in all groups at 21 days compared to the levels measured at 7 days post-irradiation.

After confirming that preconditioning with rapamycin leads to an increase in the secretion of hematopoietic factors and this effect persists in vivo, we evaluated changes in the bone marrow cellularity of the mouse femur. Bone marrow cellularity was significantly decreased at 7 and 21 days post-irradiation in the mouse femur without treatment. Transplantation of PD-MSCs or rPD-MSCs leads to an increase in cellularity of almost all hematopoietic lineages in both time points compared to mice without treatment. The use of rapamycin-preconditioned MSCs demonstrates the greatest effect on BM cellularity recovery.

Additionally, we evaluate peripheral blood cell counts in each group to prove the therapeutic effects of rapamycin-preconditioned PD-MSCs in h-ARS. Our results demonstrate that preconditioning with rapamycin leads to significant changes in peripheral blood cell count compared to non-preconditioned cells or to mice without treatment.

In conclusion, the present study reveals that placenta-derived MSCs have great potential for h-ARS treatment. Induction of mTOR-dependent autophagy by preconditioning with rapamycin could enhance the secretion of hematopoietic cytokines in PD-MSCs without affecting cell viability. Rapamycin-preconditioned cells have shown the biggest effectiveness in hematopoietic recovery, restore bone marrow cellularity, and increase peripheral blood cell counts compared to non-preconditioned cells. These results demonstrated rapamycin preconditioning as a promising strategy to enhance the MSC therapeutic potential for ARS, supporting further preclinical and clinical exploration.

## 4. Materials and Methods

### 4.1. Isolation and Culture of PD-MSCs

Placenta-derived MSCs were isolated from the chorion of outbred ICR/CD1 female mice, aged 3–4 weeks, on the 19th day of gestation. Mice were euthanized under conditions described in the “Animal care and experimental groups” section, and placental tissues were collected for further processing. Placental tissues were collected and enzymatically dissociated using Accutase (Millipore, Burlington, MA, USA, cat. № SCR005), followed by mechanical dissociation with a scalpel and pipette. The Accutase was neutralized by adding MesenCult MSC Basal Medium Mouse from MesenCult™ Expansion Kit Mouse medium (StemCell Technologies, Vancouver, BC, Canada, cat. № 05513) supplemented with 10% FBS in a 1:2 ratio. The cell suspension was centrifuged at 300× *g* for 10 min, and the pellet was resuspended in PBS. Mononuclear cells were isolated using Lympholyte^®^-M density gradient (CEDARLANE, Burlington, ON, Canada, cat. № CL5035) centrifugation at 1000× *g* for 20 min at room temperature. Cells were cultured in MesenCult MSC Basal Medium Mouse supplemented with MesenCult™ 10× Supplements Mouse, 10% FBS, 2 mmol L-glutamine (StemCell Technologies, Vancouver, BC, Canada, cat. № 07100), and antibiotics (50 U/mL penicillin, 50 µg/mL streptomycin; Sigma-Aldrich, St. Louis, MO, USA, cat. № P4333).

### 4.2. Identification of PD-MSCs by ISCT Criteria

MSCs were immunophenotyped using the Mouse Mesenchymal Stem Cell Multi-Color Flow Cytometry Kit (R&D Systems, Minneapolis, MN, USA, FMC003), according to the protocol, for determining CD105, CD29, CD45 and SCA-1 expression by flow cytometry using Beckman Coulter Navios flow cytometer (Beckman Coulter, Brea, CA, USA). Adipocytic and osteogenic differentiation were performed using the Mouse Mesenchymal Stem Cell Functional Identification Kit (R&D Systems, Minneapolis, MN, USA, SC010), according to the protocol. Adipogenic, osteogenic, and chondrogenic differentiation was established using Oil Red O (Sigma-Aldrich, St. Louis, Missouri, USA, cat. № SCR020), Alizarin Red (Sigma-Aldrich, St. Louis, Missouri, USA, cat. № 632371), and Toluidine Blue (Servicebio, Wuhan, China, cat. № G1032) staining, how it was previously described in [40], and by flow cytometry using FABP-4 and osteopontin antibodies from the Mouse Mesenchymal Stem Cell Functional Identification Kit (R&D Systems, Minneapolis, MN, USA, SC010).

### 4.3. Preconditioning of PD-MSCs

The dose of rapamycin was chosen based on previous studies [40]. For preconditioning of PD-MSCs, Rapamycin (Abcam, Cambridge, UK, cat. № ab120224) was dissolved in dimethyl sulfoxide (DMSO; Abcam, Cambridge, UK, cat. № ab146591) to prepare a stock solution. The stock solution was then added to the culture media to achieve a final concentration of 3 µM of Rapamycin. Cells were treated for 24 h under standard culture conditions.

### 4.4. Cell Viability Assay

The viability of PD-MSCs was evaluated using the cell counting kit-8 (CCK-8) assay (Servicebio, Wuhan, China, cat. № 4103-5), according to the manufacturer’s instructions. Cells were seeded into a 96-well plate, and then 10 μL of CCK-8 was added to the culture media. Two hours after incubation with CCK-8, the cells’ growth was measured. The absorbance of each well was quantified at 450 nm (Thermo Fisher Scientific Multiscan, Waltham, MA, USA). All data were calculated from triplicate samples.

### 4.5. RT-qPCR Analysis of Gene Expression

Total RNA was isolated using the ExtractRNA kit (Evrogen, Moscow, Russia, cat. № BC032). To remove genomic DNA, the isolated RNA was treated with DNase E (Evrogen Moscow, Russia, cat. № EK007S). cDNA was synthesized from the purified RNA using the REVERSE-L kit (AmpliSens, Moscow, Russia, cat. № K3-4-100) according to the manufacturer’s instructions. Quantitative RT-qPCR was performed using the HS-qPCR SYBR Blue Bio-Master (2×) master mix (Biolabmix, Novosibirsk, Russia, cat. № MHC030-400) and 10 pM of gene-specific primers. The reactions were carried out on the LOCUS^®^ Intero 6 instrument (Tianlong, Shanghai, China). GAPDH was used as an endogenous control. Each sample was analyzed in triplicate, and the relative gene expression levels were calculated using the 2^−ΔΔCt^ method [41]. The sequences of the primers used are listed in Table 1.

### 4.6. ELISA Quantification of Autophagy-Related Proteins and Hematopoietic Cytokines in Cell Lysates, Culture Media, and BM

The assessment of Beclin-1, MAP1LC3B and mTOR protein levels was carried out in MSCs lysate using an ELISA Kit for Beclin-1 (BECN1) (Cloud-Clone Corp, Wuhan, China, cat. № SEJ557Mu), ELISA Kit for Microtubule Associated Protein 1 Light Chain 3 Beta (MAP1LC3b) (Cloud-Clone Corp., Wuhan, China, cat. № SEL702Mu), and ELISA Kit for Serine/threonine-protein kinase mTOR (Cloud-Clone Corp., Wuhan, China, cat. № SEB806Mu), according to the manufacturer’s instructions.

The assessment of G-CSF, SCF, and Flt3LG protein levels was carried out in culture media and in extracellular fraction of BM using Mouse Flt3 ligand ELISA Kit (Abcam, Cambridge, UK, cat. № ab275551), Mouse G-CSF ELISA Kit (Abcam, Cambridge, UK, cat. № ab197743), and Mouse SCF ELISA Kit (Abcam, Cambridge, UK, cat. № ab197750), according to the manufacturer’s instructions.

The determination of these parameters was performed using a Chem Well 2910 enzyme immunoassay and biochemical analyzer (Combi, Charlotte, NC, USA).

### 4.7. Animal Care and Experimental Groups

All experiments were conducted on 80 outbred ICR/CD1 male mice weighing 23 ± 1 g. Mice were kept in the Ural State Medical University vivarium with a controlled environment (12 h light/dark cycle, temperature 21–22 °C, free access to water and standard rodent chow).

Mice were divided into 8 groups (*n* = 10 per group) using block randomization: (i) 7d Intact group; (ii) 7d ARS group; (iii) 7 day PD-MSC group; (iv) 7 day rPD-MSC group, (v) 21 day Intact group; (vi) 21 day ARS group; (vii) 21 day PD-MSC group; (viii) 21 day rPD-MSC group. The in vivo section of the study design is illustrated in Figure 3a.

To induce acute radiation syndrome, mice from each group (excluding the 7 day Intact group and 21 day Intact group) were irradiated using the Agat-C gamma-therapeutic unit (Russia) equipped with a Co-60 radionuclide source. A total irradiation dose of 5.0 Gy was delivered at a rate of 0.6 Gy/min. The absorbed radiation dose was monitored in real-time using a UNIDOS dosimeter coupled with a Farmer ionization chamber, which was positioned at half the height of the animals’ bodies. The irradiation process was automatically terminated once the preset dose was reached. One hour post-irradiation, mice from groups (iii), (iv), (v), and (vi) received an intravenous injection of either 1 × 10^6^ PD-MSCs or 1 × 10^6^ rapamycin-preconditioned PD-MSCs, administered via the lateral tail vein.

At the end, the mice were sacrificed under anesthesia induced by intramuscular injection of 20 mg/kg xylazine-zoletyl [42] for the collection of blood samples and bone marrow, followed by euthanasia via cervical dislocation.

### 4.8. Cytological Evaluation of Bone Marrow and Peripheral Blood Counts

Blood samples were collected via cardiac puncture under deep anesthesia to minimize animal distress. Immediately after collection, blood was transferred into pre-chilled tubes containing 1.5 mg/mL EDTA as an anticoagulant to prevent clotting. Total blood cell counts, including leukocytes, lymphocytes, granulocytes, platelets, erythrocytes, and monocytes, were quantified using a MEK6400 automated hematology analyzer (Nihon Kohden, Tokyo, Japan).

Following euthanasia by cervical dislocation, the femur was aseptically isolated using sterile surgical instruments. Both epiphyses were carefully severed, and bone marrow was flushed out using 0.5 mL of ice-cold Dulbecco’s Phosphate-Buffered Saline without calcium and magnesium (D-PBS; Pan Eco, Moscow, Russia). The resulting cell suspension was gently pipetted to ensure homogeneity and then centrifuged at 3000× *g* for 15 min at 4 °C to separate cellular components. The supernatant was discarded, and the pellet was resuspended in a small volume of D-PBS for further processing.

For morphological analysis, cytological preparations were made by spreading a drop of the bone marrow suspension onto clean glass slides. The smears were air-dried and fixed with “eosin methylene blue” dye-fixative for 2 min. Subsequently, the smears were stained using the Romanowsky method with “Azur–Eosin” (e.g., Wright-Giemsa stain) for an additional 2 min. Stained smears were rinsed with distilled water, air-dried, and mounted with a coverslip for microscopic examination. Myelogram analysis was performed by counting 1000 nucleated cells per smear under a light microscope. Cell types were classified according to standard morphological criteria, including granulocytes, erythroblasts, lymphocytes, and monocytes.

To determine the total number of cells (nucleated bone marrow cells), bone marrow was harvested from the contralateral femur using the same procedure. The cell suspension was diluted 1:20 with Türk’s solution (3% acetic acid with gentian violet) to lyse erythrocytes and facilitate counting. The total number of cells was quantified using a hemocytometer under a light microscope (100× magnification). The final cell count was expressed as the total number of cells per femur.

### 4.9. Statistical Analysis

Statistical analysis was performed using GraphPad Prism 10.0 (GraphPad Software, La Jolla, CA, USA). All values were given as means ± SD. One-way ANOVA followed by Bonferroni’s post-hoc test was used to compare the differences between three or more groups. Comparisons between two groups were performed using the Student’s *t*-test. The data were considered statistically significant at *p* < 0.05.

## Figures and Tables

**Table 1:** Oligonucleotide primers used for expression analyses.

Genes	Forward	Reverse
*GAPDH*	5′-TGTCCGTCGTGGATCTGAC-3′	5′-CCTGCTTCACCACCTTCTTG-3′
*MAP1LC3B*	5′-CCCCACCAAGATCCCAGT-3′	5′-CGCTCATGTTCACGTGGT-3′
*BECN1*	5′-AGGATGGTGTCTCTCGAAGATT-3′	5′-GATCAGAGTGAAGCTATTAGCACTTTC-3′

## Data Availability

The datasets used and/or analyzed in the current study are available from the corresponding author upon reasonable request.

## Abbreviations

The following abbreviations are used in this manuscript:

α-SMA	Alpha-Smooth Muscle Actin
ARS	Acute radiation syndrome
BM	Bone marrow
ELISA	Enzyme-Linked Immunosorbent Assay
FABP-4	Fatty Acid-Binding Protein 4
FGF-2	Fibroblast Growth Factor-2
Flt3LG	FMS-related Tyrosine Kinase 3 Ligand
G-CSF	Granulocyte Colony-Stimulating Factor
IDO	Indoleamine 2,3-Dioxygenase
IL-6	Interleukin-6
IL-10	Interleukin-10
IL-15	Interleukin-15
ISCT	International Society for Cell & Gene Therapy
LPS	Lipopolysaccharide
MAP1LC3β	Microtubule-Associated Protein 1 Light Chain 3 Beta
mTOR	Mechanistic Target of Rapamycin
PD-MSCs	Placenta-Derived Mesenchymal stem cells
SCF	Stem Cell Factor
SDF-1	Stromal Cell-Derived Factor-1
TGF-β	Transforming Growth Factor-Beta

## References

[1] Chinnadurai R., Forsberg M.H., Kink J.A., Hematti P., Capitini C.M. (2020). Use of MSCs and MSC-Educated Macrophages to Mitigate Hematopoietic Acute Radiation Syndrome. Curr. Stem Cell Rep..

[2] Rybkina V.L., Bannikova M.V., Adamova G.V., Dörr H., Scherthan H., Azizova T.V. (2018). Immunological Markers of Chronic Occupational Radiation Exposure. Health Phys..

[3] Grammaticos P., Giannoula E., Fountos G.P. (2013). Acute Radiation Syndrome and Chronic Radiation Syndrome. Hell. J. Nucl. Med..

[4] Gorbunov N.V., Sharma P. (2015). Protracted Oxidative Alterations in the Mechanism of Hematopoietic Acute Radiation Syndrome. Antioxidants.

[5] Plett P.A., Pelus L.M., Orschell C.M. (2023). Establishing a Murine Model of the Hematopoietic Acute Radiation Syndrome. Methods Mol. Biol..

[6] Samsonraj R.M., Raghunath M., Nurcombe V., Hui J.H., van Wijnen A.J., Cool S.M. (2017). Concise Review: Multifaceted Characterization of Human Mesenchymal Stem Cells for Use in Regenerative Medicine. Stem Cells Transl. Med..

[7] Eaton E.B., Varney T.R. (2015). Mesenchymal Stem Cell Therapy for Acute Radiation Syndrome: Innovative Medical Approaches in Military Medicine. Mil. Med. Res..

[8] Bandekar M., Maurya D.K., Sharma D., Sandur S.K. (2021). Preclinical Studies and Clinical Prospects of Wharton’s Jelly-Derived MSC for Treatment of Acute Radiation Syndrome. Curr. Stem Cell Rep..

[9] Rezvani M. (2021). Therapeutic Potential of Mesenchymal Stromal Cells and Extracellular Vesicles in the Treatment of Radiation Lesions—A Review. Cells.

[10] Friedenstein A.J., Petrakova K.V., Kurolesova A.I., Frolova G.P. (1968). Heterotopic of Bone Marrow. Analysis of Precursor Cells for Osteogenic and Hematopoietic Tissues. Transplantation.

[11] Lou S., Duan Y., Nie H., Cui X., Du J., Yao Y. (2021). Mesenchymal Stem Cells: Biological Characteristics and Application in Disease Therapy. Biochimie.

[12] Ripoll C.B., Bunnell B.A. (2009). Comparative Characterization of Mesenchymal Stem Cells from EGFP Transgenic and Non-Transgenic Mice. BMC Cell Biol..

[13] Dominici M., Le Blanc K., Mueller I., Slaper-Cortenbach I., Marini F., Krause D., Deans R., Keating A., Prockop D., Horwitz E. (2006). Minimal Criteria for Defining Multipotent Mesenchymal Stromal Cells. The International Society for Cellular Therapy Position Statement. Cytotherapy.

[14] de la Torre P., Flores A.I. (2021). Current Status and Future Prospects of Perinatal Stem Cells. Genes.

[15] Beeravolu N., McKee C., Alamri A., Mikhael S., Brown C., Perez-Cruet M., Chaudhry G.R. (2017). Isolation and Characterization of Mesenchymal Stromal Cells from Human Umbilical Cord and Fetal Placenta. J. Vis. Exp..

[16] Gao Y., Chi Y., Chen Y., Wang W., Li H., Zheng W., Zhu P., An J., Duan Y., Sun T. (2023). Multi-Omics Analysis of Human Mesenchymal Stem Cells Shows Cell Aging That Alters Immunomodulatory Activity through the Downregulation of PD-L1. Nat. Commun..

[17] Majumdar M.K., Thiede M.A., Mosca J.D., Moorman M., Gerson S.L. (1998). Phenotypic and Functional Comparison of Cultures of Marrow-Derived Mesenchymal Stem Cells (MSCs) and Stromal Cells. J. Cell. Physiol..

[18] Mourcin F., Grenier N., Mayol J.-F., Lataillade J.-J., Sotto J.-J., Hérodin F., Drouet M. (2005). Mesenchymal Stem Cells Support Expansion of in Vitro Irradiated CD34(+) Cells in the Presence of SCF, FLT3 Ligand, TPO and IL3: Potential Application to Autologous Cell Therapy in Accidentally Irradiated Victims. Radiat. Res..

[19] Budgude P., Kale V., Vaidya A. (2020). Mesenchymal Stromal Cell-Derived Extracellular Vesicles as Cell-Free Biologics for the Ex Vivo Expansion of Hematopoietic Stem Cells. Cell Biol. Int..

[20] Lange C., Brunswig-Spickenheier B., Reimer R., Zustin J. (2016). Mesenchymal Stromal Cells Protect from Consequences of HSCT-Transplantation Preparatory Irradiation: Insights into Possible Mechanisms. Cell. Ther. Transplant..

[21] Shim S., Lee S.B., Lee J.G., Jang W.S., Lee S.J., Park S., Lee S.S. (2013). Mitigating Effects of HUCB-MSCs on the Hematopoietic Syndrome Resulting from Total Body Irradiation. Exp. Hematol..

[22] Hu K.X., Sun Q.Y., Guo M., Ai H.S. (2010). The Radiation Protection and Therapy Effects of Mesenchymal Stem Cells in Mice with Acute Radiation Injury. Br. J. Radiol..

[23] Lange C., Brunswig-Spickenheier B., Cappallo-Obermann H., Eggert K., Gehling U.M., Rudolph C., Schlegelberger B., Cornils K., Zustin J., Spiess A.N. (2011). Radiation Rescue: Mesenchymal Stromal Cells Protect from Lethal Irradiation. PLoS ONE.

[24] Forsberg M.H., Kink J.A., Thickens A.S., Lewis B.M., Childs C.J., Hematti P., Capitini C.M. (2021). Exosomes from Primed MSCs Can Educate Monocytes as a Cellular Therapy for Hematopoietic Acute Radiation Syndrome. Stem Cell Res. Ther..

[25] Zhang C., Zhu Y., Wang J., Hou L., Li W., An H. (2019). CXCR4-Overexpressing Umbilical Cord Mesenchymal Stem Cells Enhance Protection against Radiation-Induced Lung Injury. Stem Cells Int..

[26] Kernagis D.N., Balcer-Kubiczek E., Bazyar S., Orschell C.M., Jackson I.L. (2022). Medical Countermeasures for the Hematopoietic-Subsyndrome of Acute Radiation Syndrome in Space. Life Sci. Sp. Res..

[27] Singh V.K., Seed T.M. (2021). Radiation Countermeasures for Hematopoietic Acute Radiation Syndrome: Growth Factors, Cytokines and Beyond. Int. J. Radiat. Biol..

[28] Gaberman E., Pinzur L., Levdansky L., Tsirlin M., Netzer N., Aberman Z., Gorodetsky R. (2013). Mitigation of Lethal Radiation Syndrome in Mice by Intramuscular Injection of 3D Cultured Adherent Human Placental Stromal Cells. PLoS ONE.

[29] Guo M., Dong Z., Qiao J., Yu C., Sun Q., Hu K., Liu G., Wei L., Yao B., Man Q. (2014). Severe Acute Radiation Syndrome: Treatment of a Lethally 60Co-Source Irradiated Accident Victim in China with HLA-Mismatched Peripheral Blood Stem Cell Transplantation and Mesenchymal Stem Cells. J. Radiat. Res..

[30] Fouillard L., Francois S., Bouchet S., Bensidhoum M., Elm’selmi A., Chapel A. (2013). Innovative Cell Therapy in the Treatment of Serious Adverse Events Related to Both Chemo-Radiotherapy Protocol and Acute Myeloid Leukemia Syndrome: The Infusion of Mesenchymal Stem Cells Post-Treatment Reduces Hematopoietic Toxicity and Promotes Hematopo. Curr. Pharm. Biotechnol..

[31] Qian L., Cen J. (2020). Hematopoietic Stem Cells and Mesenchymal Stromal Cells in Acute Radiation Syndrome. Oxid. Med. Cell. Longev..

[32] Dunlop E.A., Tee A.R. (2014). MTOR and Autophagy: A Dynamic Relationship Governed by Nutrients and Energy. Semin. Cell Dev. Biol..

[33] Zhang Q., Yang Y.-J., Wang H., Dong Q.-T., Wang T.-J., Qian H.-Y., Xu H. (2012). Autophagy Activation: A Novel Mechanism of Atorvastatin to Protect Mesenchymal Stem Cells from Hypoxia and Serum Deprivation via AMP-Activated Protein Kinase/Mammalian Target of Rapamycin Pathway. Stem Cells Dev..

[34] Wang B., Lin Y., Hu Y., Shan W., Liu S., Xu Y., Zhang H., Cai S., Yu X., Cai Z. (2017). MTOR Inhibition Improves the Immunomodulatory Properties of Human Bone Marrow Mesenchymal Stem Cells by Inducing COX-2 and PGE2. Stem Cell Res. Ther..

[35] Awadalla A., Hussein A.M., El-far Y.M., El-senduny F.F., Barakat N., Hamam E.T., Abdeen H.M., El-sherbiny M., Serria M.S., Sarhan A.A. (2022). Rapamycin Improves Adipose-Derived Mesenchymal Stem Cells (ADMSCs) Renoprotective Effect against Cisplatin-Induced Acute Nephrotoxicity in Rats by Inhibiting the MTOR/AKT Signaling Pathway. Biomedicines.

[36] Kim K., Moon S., Park M., Kim B., Kim E., Lee S., Lee E. (2015). Optimization of Adipose Tissue-Derived Mesenchymal Stem Cells by Rapamycin in a Murine Model of Acute Graft-versus-Host Disease. Stem Cell Res. Ther..

[37] Li Z.-H., Wang Y.-L., Wang H.-J., Wu J.-H., Tan Y.-Z. (2020). Rapamycin-Preactivated Autophagy Enhances Survival and Differentiation of Mesenchymal Stem Cells After Transplantation into Infarcted Myocardium. Stem Cell Rev. Rep..

[38] Wise R.M., Harrison M.A.A., Sullivan B.N., Al-Ghadban S., Aleman S.J., Vinluan A.T., Monaco E.R., Donato U.M., Pursell I.A., Bunnell B.A. (2020). Short-Term Rapamycin Preconditioning Diminishes Therapeutic Efficacy of Human Adipose-Derived Stem Cells in a Murine Model of Multiple Sclerosis. Cells.

[39] Gratwohl A., John L., Baldomero H., Roth J., Tichelli A., Nissen C., Lyman S.D., Wodnar-Filipowicz A. (1998). FLT-3 Ligand Provides Hematopoietic Protection from Total Body Irradiation in Rabbits. Blood.

[40] Gao L., Cen S., Wang P., Xie Z., Liu Z., Deng W., Su H., Wu X., Wang S., Li J. (2016). Autophagy Improves the Immunosuppression of CD4+ T Cells by Mesenchymal Stem Cells Through Transforming Growth Factor- β 1. Stem Cells Transl. Med..

[41] Livak K.J., Schmittgen T.D. (2001). Analysis of Relative Gene Expression Data Using Real-Time Quantitative PCR and the 2−ΔΔCT Method. Methods.

[42] Khokhlova O.N., Borozdina N.A., Sadovnikova E.S., Pakhomova I.A., Rudenko P.A., Korolkova Y.V., Kozlov S.A., Dyachenko I.A. (2022). Comparative Study of the Aftereffect of CO2 Inhalation or Tiletamine–Zolazepam–Xylazine Anesthesia on Laboratory Outbred Rats and Mice. Biomedicines.

